# Hijacking Host Cell Highways: Manipulation of the Host Actin Cytoskeleton by Obligate Intracellular Bacterial Pathogens

**DOI:** 10.3389/fcimb.2016.00107

**Published:** 2016-09-22

**Authors:** Punsiri M. Colonne, Caylin G. Winchell, Daniel E. Voth

**Affiliations:** Department of Microbiology and Immunology, University of Arkansas for Medical SciencesLittle Rock, AR, USA

**Keywords:** intracellular, bacteria, actin, cytoskeleton, pathogen

## Abstract

Intracellular bacterial pathogens replicate within eukaryotic cells and display unique adaptations that support key infection events including invasion, replication, immune evasion, and dissemination. From invasion to dissemination, all stages of the intracellular bacterial life cycle share the same three-dimensional cytosolic space containing the host cytoskeleton. For successful infection and replication, many pathogens hijack the cytoskeleton using effector proteins introduced into the host cytosol by specialized secretion systems. A subset of effectors contains eukaryotic-like motifs that mimic host proteins to exploit signaling and modify specific cytoskeletal components such as actin and microtubules. Cytoskeletal rearrangement promotes numerous events that are beneficial to the pathogen, including internalization of bacteria, structural support for bacteria-containing vacuoles, altered vesicular trafficking, actin-dependent bacterial movement, and pathogen dissemination. This review highlights a diverse group of obligate intracellular bacterial pathogens that manipulate the host cytoskeleton to thrive within eukaryotic cells and discusses underlying molecular mechanisms that promote these dynamic host-pathogen interactions.

## Introduction

Obligate intracellular bacteria comprise a group of highly infectious human pathogens that cause a spectrum of life threatening diseases. These bacteria demonstrate remarkable adaptations that support intracellular lifestyles comprised of unique challenges and advantages. To support diverse infection events, intracellular pathogens have evolved numerous methods to subvert host molecular signaling machinery that controls essential cellular functions. Intracellular pathogens display tropism for specific eukaryotic cell types and attach to these cells to initiate internalization, allowing intracellular growth using energy rich host metabolic products. Successful immune evasion is essential for productive infection, and the intracellular environment provides temporary cover from detection by immune cells and antibody/compliment-mediated immunity. Following replication, these pathogens exit the infected cell and disseminate to additional sites of infection.

The cytoskeleton is a major host structural component manipulated by intracellular pathogens to drive cellular infection. The cytoskeleton is a three-dimensional network of polymeric proteins that provides structural support and assists numerous vital cellular functions. The cytoskeletal network also functions as a highway to directionally transport cargo-containing vesicles throughout the cell. Intracellular bacteria encounter the host cytoskeletal components actin and microtubules throughout growth and have adapted to use these networks to facilitate infection. Recent studies have broadened understanding of molecular mechanisms controlling bacteria-cytoskeleton interactions and their impact on cellular infection. Here, we focus on cytoskeletal manipulation by obligate intracellular bacterial pathogens generally considered non-cultivatable *in vitro*. These human pathogens include *Chlamydia* (major cause of sexually transmitted disease), *Rickettsia* (Rocky Mountain Spotted Fever, Mediterranean Spotted Fever, and epidemic typhus), *Anaplasma* (human granulocytic anaplasmosis), and *Ehrlichia* (human monocytic ehrlichiosis) species. We also include the Q fever agent *Coxiella burnetii*, which replicates exclusively within host cells during natural infection, but can be cultured in specialized axenic media *in vitro*. The interaction of these pathogens with the host cytoskeletal network has been studied for many years, but detailed molecular mechanisms are lacking due to difficulty in genetically manipulating these bacteria. However, recent genetic breakthroughs provide tractable systems by which bacteria-actin interactions can now be defined for this intriguing group of pathogens (Beare et al., 2009, 2012; Burkhardt et al., 2011; Clark et al., 2011; Wood et al., 2012; Cheng et al., 2013; Crosby et al., 2014). We highlight how each pathogen hijacks host cytoskeletal machinery to facilitate infection events including invasion, intracellular replication, and dissemination.

## Mammalian cell highways: multi-functional dynamic cytoskeletal networks

The mammalian cytoskeleton consists of three major components: microfilaments, microtubules, and intermediate filaments. Microfilaments are composed of globular (G)-actin that hydrolyzes ATP to provide energy for F-actin polymerization (Reisler, 1993; Graceffa and Dominguez, 2003). Actin nucleation is the assembly of actin monomers into short multimers and requires nucleating factors such as actin related protein 2/3 (Arp2/3) and formins. The Arp 2/3 complex consists of seven evolutionarily conserved subunits that bind to Wiskott-Aldrich Syndrome (WASP) proteins to initiate nucleation. (Egile et al., 2005; Pollard, 2007). Additional proteins such as profilin, thymosin, and cofilin, are also directly involved in regulating actin polymerization (Carlsson et al., 1977; Goldschmidt-Clermont et al., 1992). Mammalian cells respond to extracellular stimuli by rearranging the cytoskeleton via activity of intracellular signaling cascades. The Rho family of small GTP-binding proteins, including Rho, Rac, and Cdc42, regulates actin microfilament dynamics (Ridley, 2006; Bustelo et al., 2007). Rho proteins are activated by phosphorylation and activate downstream target proteins to control actin polymerization. Microtubules are hetero-polymers composed of α- and β-tubulin, and microtubule organizing centers (MTOCs) provide a platform for assembly of the asymmetric microtubule network (Vinogradova et al., 2009). Microfilaments and microtubules serve multifunctional roles in processes such as cell migration and intracellular vesicular trafficking, and provide structural support for the cell. Intermediate filaments are formed by many proteins, including the most widely distributed filament protein vimentin. Intermediate filaments perform several structural functions including anchoring organelles in the cytoplasm and maintaining cell shape and integrity (Kirmse et al., 2007; Nekrasova et al., 2011).

## Intruder alert: using rapid actin polymerization to invade host cells

**Figure 1:**
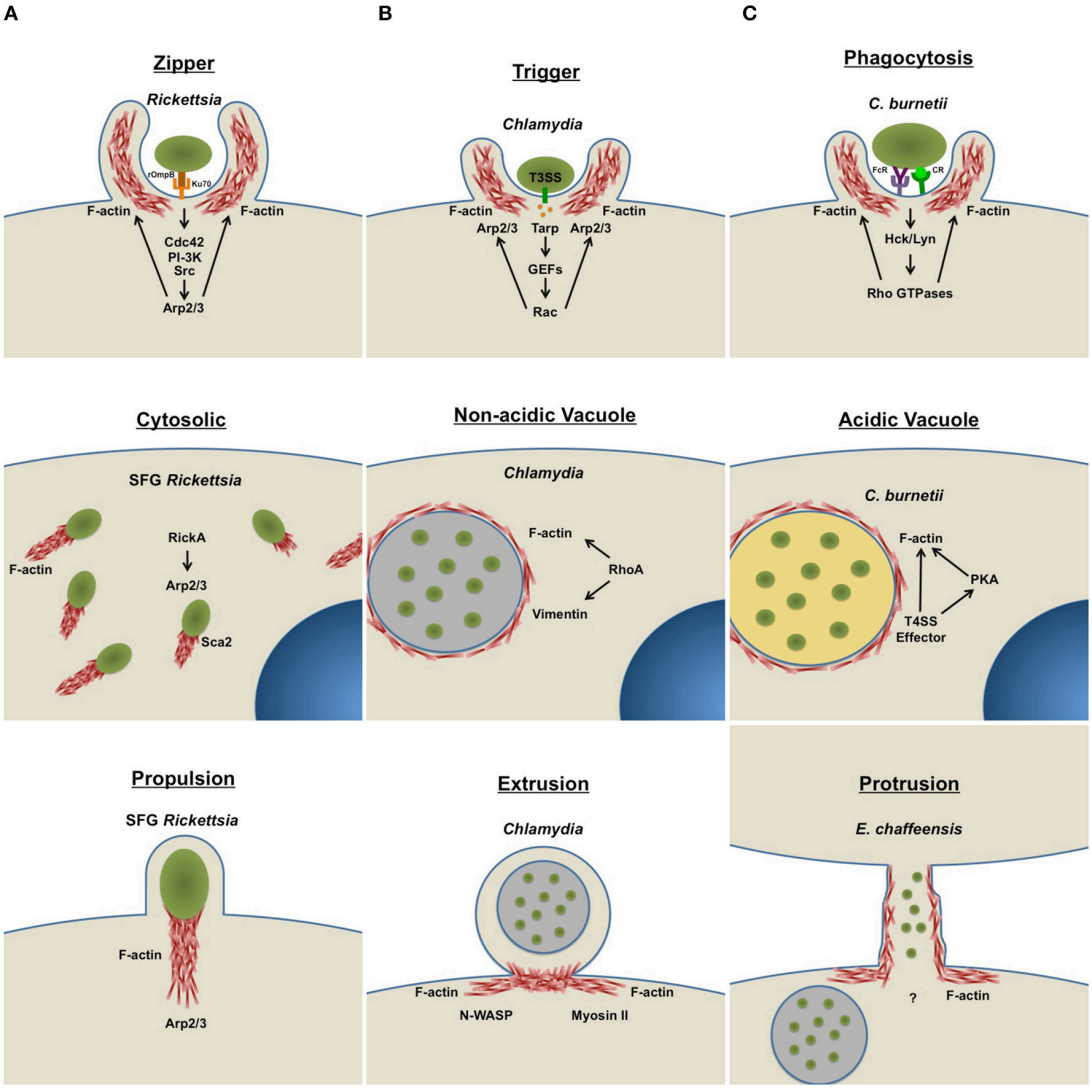
**Intracellular bacteria use host actin for diverse infection events**. Intracellular bacteria actively manipulate the host actin cytoskeleton to facilitate major lifecycle events, such as cellular invasion, intracellular replication, and dissemination. Arrows indicate activation of specific host cell components. **(A)** Host cell entry: In non-phagocytic cells, bacteria use a zipper (*Rickettsia*) or trigger (*Chlamydia*) mechanism to facilitate infection. Phagocytic cells engulf bacterial pathogens (*C. burnetii* and *E. chaffeensis*) by phagocytosis. To promote uptake, bacteria actively remodel actin at the attachment site by activating host kinase signaling cascades. **(B)** Intracellular life: Some cytosolic bacteria (SFG *Rickettsia*) form actin tails to facilitate mobility within the cell. Pathogens that reside within membrane-bound vacuoles (*Chlamydia, C. burnetii*, and *E. chaffeensis*) manipulate the cytoskeleton to facilitate vacuole formation and stability. **(C)** Host cell escape: One group of bacteria (SFG *Rickettsia*) use actin-based motility to exit infected cells and move into non-infected bystander cells. Some host membrane-bound pathogen-containing vacuoles are pinched off by extrusion (*Chlamydia*), a process that requires actin remodeling. Other intracellular bacteria actively remodel actin at the host cell surface to form membrane protrusions (*E. chaffeensis*), connecting infected and uninfected neighboring cells. Bacteria are then transported through protrusions into neighboring cells to start a new infection cycle. T3SS, type III secretion system; T4SS, type IV secretion system; FcR, Fc Receptor; CR, Complement Receptor.

Host cell entry is the first step in the intracellular pathogen invasion process. Bacterial cell wall components facilitate attachment to specific host cells via distinct receptors. Pathogens use the rearranging abilities of actin to facilitate rapid entry into the host cell, with phagocytic and non-phagocytic cells internalizing bacteria by different mechanisms (Figure 1A). Bacterial cell surface antigen interaction with phagocytic cell receptors activates actin rearrangement to form membrane protrusions and internalize the pathogen (Carabeo et al., 2002; Martinez and Cossart, 2004; Martinez et al., 2005; Rosales et al., 2012). Non-phagocytic cells internalize bacteria by “zipper” or “trigger” mechanisms activated by the pathogen. Cytoskeletal rearrangement at the bacterial attachment site is a conserved feature of entry in phagocytic and non-phagocytic cells. Actin rearrangement is triggered by activation of host kinase signaling and pathogens target distinct kinases. The zipper mechanism occurs following interaction of a bacterial protein with a host cell receptor, triggering intracellular signaling via activation of adaptor proteins and kinases, and stimulating filamentous actin rearrangement and endocytosis. The trigger method is mechanically and morphologically distinct from the zipper strategy and initiates when bacteria inject effector proteins into the host cell using a specialized secretion system. These bacterial effector molecules activate host GTPases and promote cytoskeletal rearrangement to trigger internalization (Alonso and Garcia-del Portillo, 2004).

*C. burnetii* preferentially infects phagocytic human macrophages via binding to CR3 receptors, triggering reorganization of filamentous actin at the attachment site (Meconi et al., 1998). Cytoskeletal ruffling is stimulated by activation of Src tyrosine kinases such as haemopoietic cell kinase (Hck) and Lyn. Hck phosphorylates WASP and regulates WASP-dependent actin polymerization (Shi et al., 2009). Inhibition of Src activity prevents actin ruffling and *C. burnetii* entry into host cells (Meconi et al., 2001), and Rho GTPases regulate internalization into phagocytic and non-phagocytic cells. Expression of dominant negative mutants or siRNA-mediated silencing of RhoA, Rac1, and Cdc42, significantly reduces *C. burnetii* entry into host cells (Salinas et al., 2015). Rho GTPases may directly control internalization by regulating actin remodeling at the bacterial attachment site, and RhoA effector proteins such as mDia1 and ROCK are also required for entry. Additionally, the actin regulator cortactin plays a role in *C. burnetii* entry into non-phagocytic cells. The cortactin SH3 domain and serine phosphorylation are required for efficient internalization and active cortactin binds F-actin to facilitate recruitment of Arp 2/3 (Rosales et al., 2012). However, it is not known if cortactin functions in *C. burnetii* internalization by phagocytic cells (Weed et al., 2000; Daly, 2004).

*Rickettsia* spp. use a zipper-like mechanism termed “induced phagocytosis” to invade non-phagocytic cells (Walker and Winkler, 1978; Walker, 1984). *R. conorii* invasion requires actin rearrangement via recruitment and activation of Arp2/3 (Martinez and Cossart, 2004) following interaction with the host cell receptor Ku70 (Martinez et al., 2005). This event is triggered by rickettsial rOmpB binding to host Ku70, activating intracellular signaling. Additionally, Src, PI-3 kinase (PI-3K), and Cdc42 activity are required for bacterial internalization (Martinez and Cossart, 2004), and PI-3K and Src are known regulators of Arp2/3 activity. The Src family member C-Src and cortactin localize to the bacterial entry site. Cdc42 is also recruited to the entry site and activates Arp2/3 to regulate actin polymerization via direct binding to WASP proteins (Higgs and Pollard, 2001). Interplay between these kinases regulates Arp2/3 activation and cytoskeleton rearrangement at the bacterial attachment site, allowing bacterial internalization.

*C. trachomatis* enters non-phagocytic cells by a “trigger” mechanism. Delivery of proteins into the host cell via a type III secretion system is essential for bacterial entry (Muschiol et al., 2006; Wolf et al., 2006), and translocated actin recruiting phosphoprotein (Tarp) is a secreted protein directly involved in cytoskeletal remodeling. Tarp contains an actin binding domain that promotes actin nucleation and a proline rich domain involved in nucleation of new filaments (Jewett et al., 2006). Upon entering the host cytosol, Tarp is phosphorylated and acts as a scaffold for binding to host proteins such as Sos1 and Vav2, known Rac guanine nucleotide exchange factors (GEFs) (Lane et al., 2008). These GEFs activate Rac GTPases required for actin rearrangement (Carabeo et al., 2004), promoting Arp2/3-dependent actin recruitment to the site of invasion. Actin rearrangement also promotes formation of pedestal-like structures, leading to bacterial internalization into membrane bound vesicles (Carabeo et al., 2002).

Interactions between the tick-borne pathogen *A. phagocytophilum* and the cytoskeleton have been largely studied in tick cells. However, only limited information is available regarding function of the tick cell cytoskeleton in pathogen infection. Altered actin dynamics in tick cells have been observed following *A. phagocytophilum* invasion and are implicated as a mechanism for intracellular survival rather than entry (Sultana et al., 2010). Indeed, *A. phagocytophilum* infection triggers actin phosphorylation and inhibits actin polymerization, increasing the presence of nuclear G-actin and inducing expression of *salp16* that supports bacterial survival (Sultana et al., 2010). Cytoskeletal rearrangement is also important during *A. phagocytophilum* invasion of neutrophils. Treatment with the actin polymerization inhibitor cytochalasin D prevents *A. phagocytophilum* entry into HL-60 cells, a model of neutrophil infection (IJdo et al., 2007). *A. phagocytophilum* binding to neutrophil P-selectin glycoprotein ligand 1 (PSGL-1) is essential for invasion (Herron et al., 2000; Sarkar et al., 2007), and PSGL-1 signaling activates Syk, followed by activation of the kinase ROCK-1. Syk depletion or blocking PSGL-1 activation inhibits ROCK-1 phosphorylation and prevents bacterial entry (Carlyon et al., 2003). ROCK-1 regulates actin remodeling, and ROCK-1-dependent cytoskeletal rearrangements may be required for bacterial invasion. However, *A. phagocytophilum*-infected primary human neutrophils do not display substantial actin rearrangement. Thus, further studies are needed to delineate the role of cytoskeletal remodeling in *A. phagocytophilum* invasion of neutrophils.

*E. chaffeensis* targets human monocytes/macrophages for replication within the host. *E. chaffeensis* triggers internalization using the invasin EtpE, which binds to the host glycosylphosphatidylinositol (GPI)-anchored protein DNase X located on the cell surface. This interaction triggers cytoskeletal rearrangement and filopodia formation (Moumène et al., 2015). CD147 interacts with the EtpE-DNase X complex and is recruited to entry foci (Mohan Kumar et al., 2015). Heterogeneous nuclear ribonucleoprotein K also interacts with CD147 and N-WASP, which is recruited to entry foci and triggers actin rearrangement required for bacterial entry. Additionally, a recent study demonstrated the importance of host Wnt signaling in *E. chaffeensis* uptake by macropahges. Luo et al showed that ehrlichial TRP120 stimulates phagocytosis and requires activation of the Wnt pathway (Luo et al., 2016), establishing a link between an *E. chaffeensis* effector and host cell uptake.

## Hide or ride: cytoskeletal remodeling to build an intracellular home

After entering a host cell, bacteria encounter a potentially hostile environment. Internalized bacteria are subjected to host cell responses including phagolysosomal degradation and exposure to reactive oxygen species. Intracellular pathogens have evolved methods to subvert these responses and create a niche that allows replication (Figure 1B). However, one method is often insufficient, and pathogens hijack many signaling pathways to manipulate the host cellular response and cytoskeleton.

After entering a host cell, *Rickettsia* escape the phagosome before lysosomal fusion. Failure to escape results in bacterial degradation in a phagolysosome. After escape, Spotted Fever Group *Rickettsia*, such as *R. rickettsii* and *R. conorii*, stimulate actin polymerization to form an actin tail that provides bacterial motility within the cell. This motility occurs in two independent phases dictated by the rickettsial protein involved. RickA localizes to the bacterial pole and facilitates actin polymerization that promotes early slow movement of bacteria through the cytoplasm shortly after uptake into a eukaryotic cell. RickA contains a WASP homology 2 (WH2) domain that binds to actin monomers and WASP proteins. RickA ultimately activates the Arp2/3 complex, promoting actin polymerization and intracellular motility (Gouin et al., 2004; Jeng et al., 2004). Later during infection, the rickettsial autotransporter Sca2, which resembles host formin homology proteins, is required for actin tail formation that promotes fast, directional motility (Haglund et al., 2010; Kleba et al., 2010; Madasu et al., 2013). This faster mode of movement is independent of RickA and Arp2/3 and requires polar localization of Sca2 (Reed et al., 2014). Members of the Typhus Group *Rickettsia*, such as *R. prowazekii*, do not form actin tails but replicate in the host cytosol following phagosomal escape (Silverman et al., 1980; Winkler and Turco, 1988).

*Chlamydia* replicate within host membrane-derived compartments termed inclusions. Following invasion of a host cell, nascent *Chlamydia* elementary bodies are trafficked toward the Golgi apparatus and perinuclear region near the minus end of microtubules, aggregating at the MTOC (Grieshaber et al., 2003). Inclusion development requires intracellular trafficking, and cytoskeletal interactions are necessary for optimal inclusion formation. Actin, microtubules, and intermediate filaments organize around the inclusion, forming a cytoskeletal cage (Campbell et al., 1989; Kumar and Valdivia, 2008). Compact, uniform F-actin rings surround inclusions and are essential to maintain vacuole morphology and integrity. RhoA, but not Rac1 or Cdc42, is required for actin assembly around the inclusion. Additionally, intermediate filament assembly requires Rho-mediated F-actin assembly to stabilize inclusions (Kumar and Valdivia, 2008).

*E. chaffeensis, A. phagocytophilum*, and *C. burnetii* also replicate within host membrane-derived vacuoles. *E. chaffeensis* may manipulate the cytoskeleton through SUMOylation-dependent protein-protein interactions between bacterial effectors and host cytoskeletal components. During invasion, the *E. chaffeensis* protein TRP120 is secreted into the host cell cytosol via a type I secretion system and is SUMOylated by host proteins. SUMOylated TRP120 interacts with γ-actin and the myosin component Myo10. Manipulation of Myo10, which is involved in microtubule cargo trafficking, may direct a supply of nutrients to expanding *E. chaffeensis* vacuoles, promoting bacterial replication (Dunphy et al., 2014).

During biogenesis of the *A. phagocytophilum*-containing vacuole, the intermediate filament vimentin is organized around the vacuole, potentially providing structural stability to the compartment (Sukumaran et al., 2011). Mechanisms that control vimentin rearrangement around the vacuole have not been characterized. However, the *A. phagocytophilum* protein AptA directly interacts with vimentin and localizes around inclusions (Sukumaran et al., 2011). AptA activates host Erk1/2, and reorganization of vimentin around inclusions is essential for kinase activation. Moreover, vimentin and Erk1/2 activation are required for optimal *A. phagocytophilum* growth in host cells (Sukumaran et al., 2011), indicating a critical role for intermediate filaments in *A. phagocytophilum* infection.

*C. burnetii* replicates within an acidic compartment termed the parasitophorous vacuole (PV) in macrophages. F-actin assembles around the PV and is required for optimal vacuole formation (Aguilera et al., 2009). However, detailed mechanisms that regulate actin dynamics around the PV have not been fully characterized. Studies in our laboratory demonstrated that *C. burnetii* hijacks cAMP-dependent protein kinase (PKA) signaling using a type IV secretion system, and this process is essential for PV formation (MacDonald et al., 2012; Macdonald et al., 2014). Ongoing studies are investigating a possible role for PKA activity in altering F-actin dynamics around the PV.

## Coming out of hiding: pathogen host cell exit strategies

Although creating a replication niche within host cells provides temporary cover from host immune defenses, bacterial pathogens have also developed exit strategies (Figure 1C) to disseminate within the host. The host cytoskeletal network that provides structural rigidity near the plasma membrane may interfere with bacterial exit. Obligate intracellular pathogens can exit host cells by cell-to-cell spread, host cell lysis, or extrusion of bacteria-containing compartments (Hybiske and Stephens, 2007, 2008; Thomas et al., 2010). The success of these exit strategies depends on efficient actin rearrangement. Some intracellular bacteria exit by host cell lysis and cell-to-cell spreading mechanisms. However, host cell lysis directly releases large numbers of bacteria and host cytosolic components into the bloodstream, potentially triggering an immune response, and necessitating cell-to-cell spread to disseminate with minimal detection.

Spotted Fever group *Rickettsia* use actin tails for propulsion into filopodia where bacteria can be released extracellularly into the bloodstream or enter adjacent cells (Van Kirk et al., 2000; Heinzen, 2003). Actin-mediated propulsion provides a mechanism for pathogen spread from cell-to-cell without exposure to host immune cells. However, exit of *Rickettsia* through host cell membranes causes endothelial cell damage and vascular leakage, resulting in characteristic pathologies (i.e., rash) observed in infected patients. Typhus group *Rickettsia* do not form actin tails (Heinzen et al., 1993), but replicate to high numbers in endothelial cells until the cell ruptures and bacteria disseminate in the bloodstream.

*E. chaffeensis* uses two different exit strategies during early and late stages of infection (Thomas et al., 2010). During early stages, infected host cells form protrusions consisting of filopodia. *E. chaffeensis* is transported into filopodia that extend into neighboring cells, providing a direct path for bacterial entry into nearby cells without immune detection. Actin rearrangement in infected cells is essential for filopodia formation and bacterial localization into filopodia. However, during late stages of infection, bacteria exit following host cell lysis (Thomas et al., 2010). Regarding *Anaplasma*, it has been suggested that *A. phagocytophilum* exits host granulocytes by exocytosis and host cell lysis. However, specific roles for cytoskeletal components during pathogen exit have not been characterized (Rikihisa, 2010).

*Chlamydia* can exit host cells by host cell lysis or extrusion of the inclusion. Extrusion requires actin rearrangement and is considered an exocytosis-like process. Extrusion initiates with formation of inclusion-containing protrusions from infected cells. Extrusions are then pinched into separable compartments located at the cell periphery, allowing inclusion release without triggering cell death (Todd and Caldwell, 1985; Hybiske and Stephens, 2007). Inhibition of actin polymerization and N-WASP activity completely prevents extrusion, while preventing microtubule formation does not impact extrusion. Inhibition of myosin II, an ATP-dependent motor protein that interacts with actin filaments, also prevents extrusion. Inhibition of Rho GTPases does not impact protrusion formation, but arrests extrusion at the pinching step (Todd and Caldwell, 1985; Hybiske and Stephens, 2007).

## Conclusions

Obligate intracellular bacteria include a unique group of human pathogens that have adapted to thrive within the host cell environment. These pathogens hijack host cell signaling by secreting bacterial effector proteins into the host cytosol to promote formation of a replication-permissive niche. Intracellular bacteria constantly encounter the dynamic host cytoskeletal network and have evolved methods to use actin and related proteins to facilitate infection. Intracellular pathogens rearrange the actin cytoskeleton during internalization by phagocytic and non-phagocytic host cells. Some intracellular bacteria replicate within a specialized membrane-bound vacuole, while others replicate free in the cytosol. Host cytoskeletal proteins often form a filamentous cage around bacteria-containing vacuoles, providing structural support and allowing vesicle fusion with the vacuole. Other intracellular bacteria stimulate actin polymerization and form actin tails that aid bacterial movement within the host cytosol and propulsion into neighboring cells. Intracellular bacteria further manipulate the actin cytoskeleton to exit host cells by lysis or vacuole extrusion. In conclusion, major events in bacterial lifecycles require host cytoskeletal components, and blocking these interactions negatively impacts bacterial replication. However, we clearly have more to learn about the molecular mechanisms controlling pathogen interaction with the host cytoskeleton. Targeting these interactions may provide novel approaches to develop antibacterial therapeutics targeting obligate intracellular bacterial pathogens.

## Author contributions

All authors listed, have made substantial, direct and intellectual contribution to the work, and approved it for publication.

### Conflict of interest statement

The authors declare that the research was conducted in the absence of any commercial or financial relationships that could be construed as a potential conflict of interest.
